# Neuronal microRNA regulation in Experimental Autoimmune Encephalomyelitis

**DOI:** 10.1038/s41598-018-31542-y

**Published:** 2018-09-07

**Authors:** Camille A. Juźwik, Sienna Drake, Marc-André Lécuyer, Radia Marie Johnson, Barbara Morquette, Yang Zhang, Marc Charabati, Selena M. Sagan, Amit Bar-Or, Alexandre Prat, Alyson E. Fournier

**Affiliations:** 1 McGill University, Montréal Neurological Institute, Montréal, QC H3A 2B4 Canada; 2 Centre de Recherche du Centre Hospitalier de l’Université de Montréal, Université de Montréal, Montréal, QC H2X 0A9 Canada; 3 McGill University, Goodman Cancer Research Centre, Montréal, H3A 1A3 Canada; 4 McGill University, Departments of Microbiology & Immunology and Biochemistry, Montréal, QC H3G 0B1 Canada; 5 Perelman School of Medicine, University of Pennsylvania, Philadelphia, PA 19104 USA

## Abstract

Multiple sclerosis (MS) is an autoimmune, neurodegenerative disease but the molecular mechanisms underlying neurodegenerative aspects of the disease are poorly understood. microRNAs (miRNAs) are powerful regulators of gene expression that regulate numerous mRNAs simultaneously and can thus regulate programs of gene expression. Here, we describe miRNA expression in neurons captured from mice subjected to experimental autoimmune encephalomyelitis (EAE), a model of central nervous system (CNS) inflammation. Lumbar motor neurons and retinal neurons were laser captured from EAE mice and miRNA expression was assessed by next-generation sequencing and validated by qPCR. We describe 14 miRNAs that are differentially regulated in both neuronal subtypes and determine putative mRNA targets though *in silico* analysis. Several upregulated neuronal miRNAs are predicted to target pathways that could mediate repair and regeneration during EAE. This work identifies miRNAs that are affected by inflammation and suggests novel candidates that may be targeted to improve neuroprotection in the context of pathological inflammation.

## Introduction

Multiple sclerosis (MS) is an autoimmune neurodegenerative disease characterized by the infiltration of peripherally activated immune cells into the central nervous system (CNS)^1^. Inflammation results in multiple foci of oligodendrocyte damage and demyelination throughout the CNS including the cerebral cortex, spinal cord, and optic nerve^2,3^. Active lesions also contain axonal ovoids, indicative of newly damaged axons, and extensive accumulation of amyloid precursor protein (APP) resulting from impaired axonal transport^4^. Axonal transection is apparent early in the disease and is thought to be a major cause of persistent neurological disability^2,4^. Gray matter atrophy and cortical thinning, as a result of neuronal death, also increase with MS-related disability, and are characteristic of the progressive forms of MS^5–7^.

In experimental autoimmune encephalomyelitis (EAE), an animal model of CNS inflammation induced by active sensitization with myelin antigens, foci of demyelination and axon damage are also apparent^8^. Infiltration begins at the lumbar region of the spinal cord and progresses anteriorly resulting in ascending paralysis. Evidence of sub-lethal neuronal damage includes dendrite shortening, thinning and fragmentation while axons display accumulations of APP and transections^9–11^. Significant loss of α- and γ-motor neurons in lumbar spinal cord has also been reported over the course of the disease, beginning as early as 14 days post immunization (dpi)^11^. Similarly, the visual system is affected in EAE with loss of retinal ganglion cells (RGCs) and optic nerve pathology including immune cell infiltration, demyelination and glial activation^12–14^. The pathology is delayed in the optic nerve relative to the spinal cord, with loss of RGCs occurring after the peak stage, around 35 dpi^13^.

The molecular changes occurring within neurons in EAE and MS that mediate pathological responses to inflammation have not been fully characterized. Understanding the molecular response to damage will be critical to devising neuroprotective strategies to mitigate sub-lethal axonal damage and neuronal cell death. We sought to identify conserved molecular networks that regulate the response to injury in all neuronal subtypes by profiling neuronal microRNAs (miRNAs) in affected neuronal subtypes. miRNAs are short RNA molecules approximately 22 nucleotides in length that bind to target messenger RNAs (mRNAs) to regulate hubs of gene expression^15^. A single miRNA can target several different mRNAs, and several miRNAs often work cooperatively to target a single mRNA^15,16^. Altered miRNA expression has been identified in the blood cells of MS patients, in active and inactive MS lesions, and in immune cell subsets and oligodendrocytes in EAE, but neuronal miRNAs have not been profiled^17–23^. Here, we describe miRNA regulation in lumbar motor neurons and retinal neurons collected from mice afflicted with EAE. mRNA targets of regulated miRNAs were assessed by *in silico* approaches. We describe considerable overlap in miRNA regulation between lumbar motor neurons and retinal neurons derived from EAE mice suggesting conserved responses to inflammation. We also identify several molecular pathways that are predicted targets of regulated miRNAs, with confirmed regulation of representative genes of these pathways, suggesting novel avenues of investigation to mediate neuroprotection in the context of inflammation.

## Results

### Differential miRNA expression over the course of EAE in lumbar motor neurons

**Figure 1:**
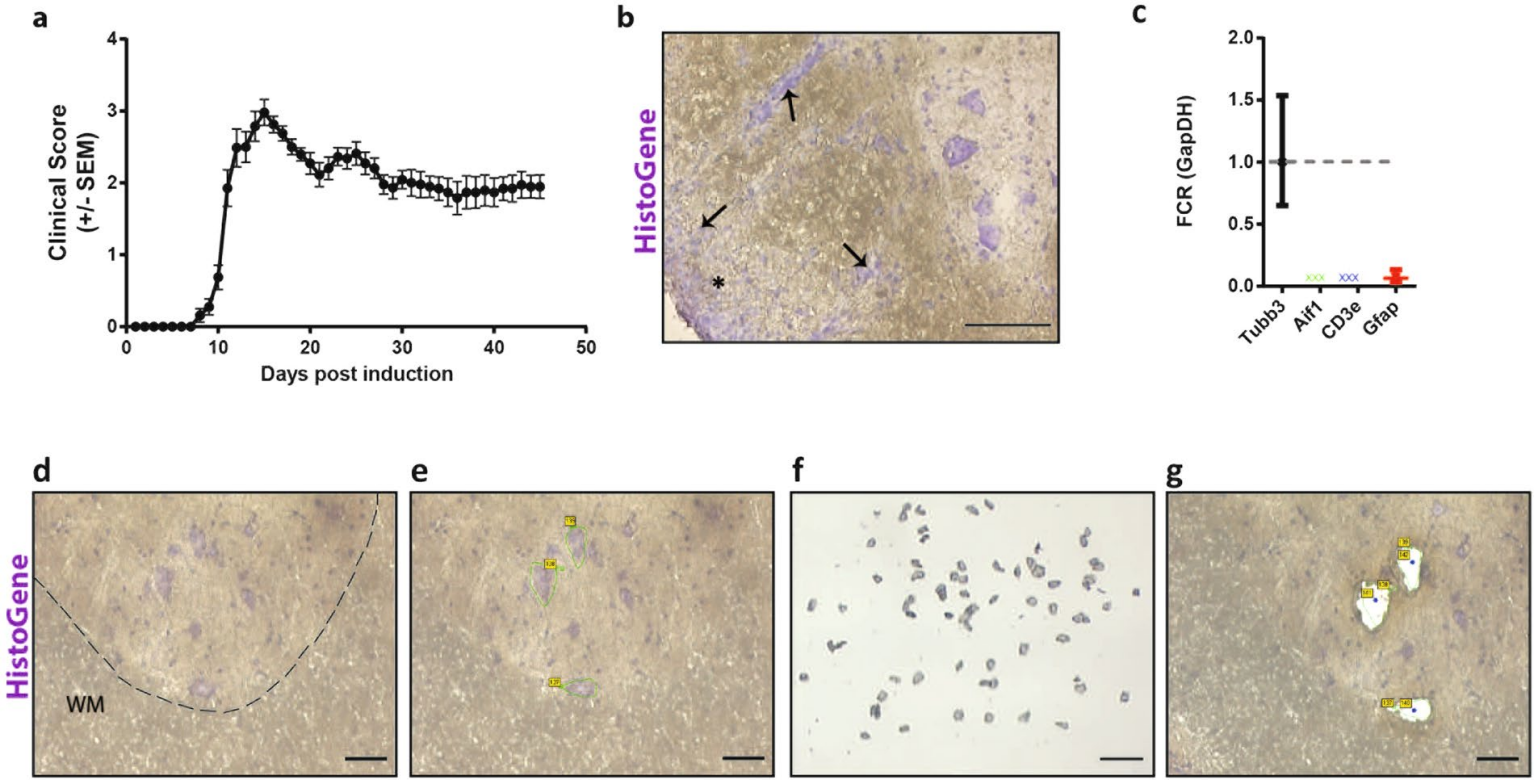
Laser captured lumbar motor neurons from EAE and control naive mice. (**a**) Clinical scores of mice immunized with MOG_35–50_ peptide. Animals of interest were taken at onset (score 0.5 to 1) and peak (score 3–3.5) of disease. Curve represents a cohort of 38 immunized animals, with representative animals taken at onset and peak. (**b**) Representative EAE spinal cord section from an animal at peak disease (score 3–3.5) stained with HistoGene and displaying immune cell infiltrates (arrows) and areas of demyelination (*) (scale bar at 100 um). (**c**) qPCR of LCM motor neuron tissue for microglia/macrophages (Aif1), immune cells (Cd3e), and astrocytes (Gfap) relative to neuronal RNA expression (Tubb3). FCR, Fold Change Range. (**d–g**) Micrographs of the laser capture microdissection flow-through. Sections of frozen mouse spinal cord on PEN membrane slides stained with HistoGene. Area above the dotted line is the dorsal horn and the area below is the white matter, WM. (**d**) The tissue surrounding the lumbar motor neurons was traced and ablated loosening the neuron of interest. (**e**) The neuron was then catapulted into an adhesive cap (**f**) leaving a void on the slide. (**g**) Scale bar; 50 um (**d**,**e**,**g**) 250 um (**f**).

**Figure 2:**
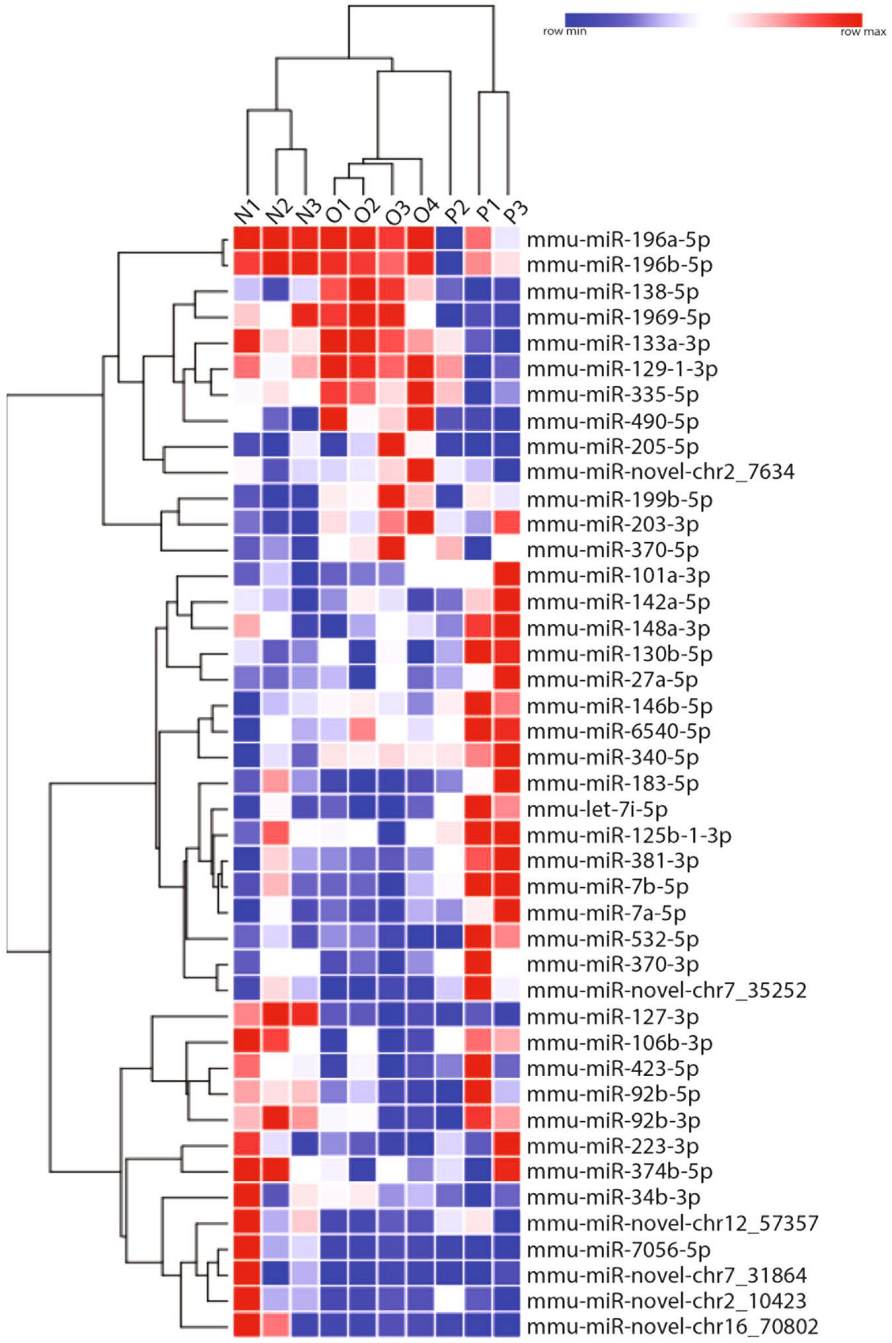
Heat Map summary of significantly regulated miRNA as identified by miR-Seq in EAE. miRNA and animals (N = naive, O = onset, P = peak) are hierarchically clustered by Euclidean distance using rlog transformed counts. Blue data represents low expression of that microRNA within its own row, and red indicates high expression. 997 miRNAs were identified by miR-Seq, 43 of these were identified as significantly regulated (p < 0.05); 6 of which are novel miRNAs.

To assess neuronal miRNA expression in response to neuroinflammation we isolated lumbar motor neurons of EAE mice over the course of their disease and profiled miRNA expression by miR-Seq NGS. EAE was induced in C57Bl/6 mice by immunization with MOG_35–55_ in CFA. EAE mice were sacrificed at three time points: naïve (score 0), onset (score 0.5–1) and peak (score 3–3.5) (Fig. 1a). Demyelination and immune cell infiltration were visible within the lumbar spinal cord by HistoGene stain at peak disease (Fig. 1b). Lumbar motor neurons were isolated from sections of fresh frozen spinal cord tissue by laser capture microdissection (LCM) (Fig. 1d–g). Neuronal purity of total RNA from the LCM tissue was confirmed by assessing the levels of glial cell and immune cell RNA markers relative to neuron specific Tubb3 levels (Fig. 1c). The efficacy of individual probes was validated on purified immune cells and astrocytes (Supplementary Table S1). We focused on the lumbar motor neurons of the spinal cord because the disease pathology of EAE begins in the lumbar region with a limp tail (score 1) and ascends the spinal cord with loss of hindlimb function (score 2–3), until mice are moribund (score 5)^24^. By collecting lumbar motor neurons at onset, we captured miRNA changes at a time when APP-positive ovoids were present but prior to significant motor neuron loss^11^. Total extracted RNA was used to prepare miRNA sequencing libraries and sequenced on Illumina HiSeq. The rlog transformed counts for miRNAs identified as significantly regulated by DeSeq2 are depicted in Fig. 2, where we can see that the animals for each EAE disease stage cluster most closely together (n = 3–4 per animal group). From the miR-Seq data, 997 miRNAs were identified, 43 of which were differentially regulated. Six of the miRNAs were novel miRNAs.

**Figure 3:**
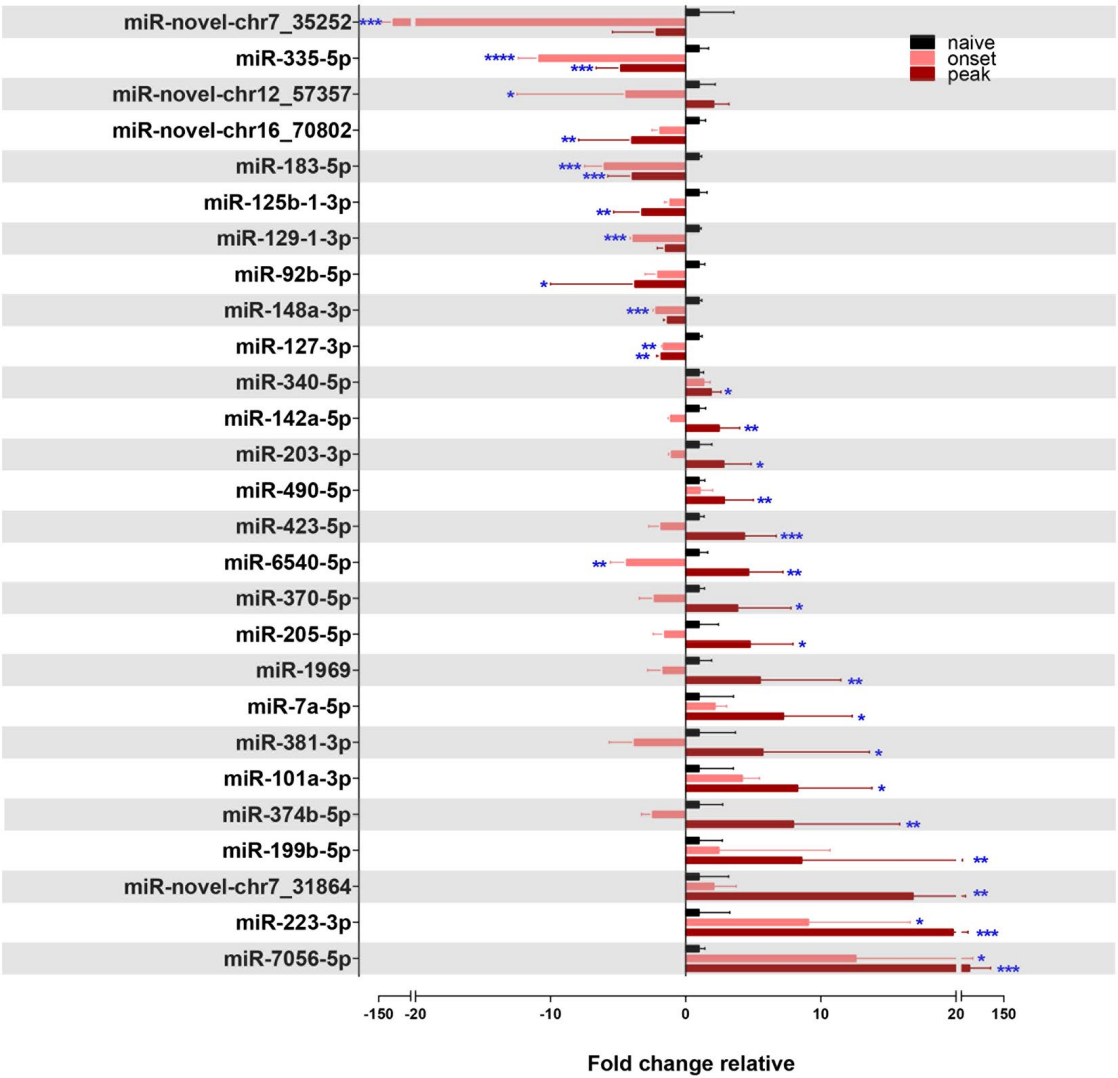
Differentially regulated miRNAs in the lumbar motor neurons of EAE mice over the course of the disease. Taqman MicroRNA Assay (qPCR) validation of miR-Seq identified differentially regulated miRNAs in the lumbar motor neurons of EAE mice over the course of the disease, normalized to endogenous control snoRNA202 for each individual miRNA and depicted as fold change relative to normalized naive levels. ****p < 0.0001, ***p < 0.001, p** < 0.01, *p < 0.05 (n = 3–6, one-way ANOVA, p < 0.05, Dunnett’s multiple comparisons test).

To validate the miR-Seq data, miRNA expression was analyzed using Taqman MicroRNA Assays. For the novel miRNAs and mmu-miR-92b-3p, custom Taqman MicroRNA Assays were designed (Supplementary Table S2). Probes for miR-92b-3p and miR-novel-chr2_10423 failed quality control testing thus qPCR validation was performed for 41 of the 43 regulated miRNAs. The fold change miRNA expression was relative to naïve levels, after normalization to endogenous control snoRNA202, using the 2^−ΔΔCt^ method^25^. Of the 41 miRNAs significantly regulated by miR-Seq, 24 were significantly regulated similarly between the miR-Seq and qPCR analyses (Fig. 3 and Table 1). Three miRNAs, miR-1969, miR-7056-5p, and miR-novel-chr7_31864, were regulated in the opposite direction compared to the miR-Seq analysis. These miRNAs carry GC rich areas which create an alignment issue during NGS, making quality reads in those regions more difficult to produce^26^. Low quality reads for a miRNA are discarded during the NGS process; this alignment issue is reduced with increased amounts of total RNA. As our total RNA was collected from LCM material, we were able to submit approximately 300 ng per biological replicate, this runs a risk of lower sequencing read counts, lower detectable rates and higher false discovery rates. Some miRNAs (specifically miR-335-5p, miR-183-5p, miR-125b-1-3p, miR-129-1-3p, miR-490-5p, miR-6540-5p, miR-205-5p, and miR-223-3p) showed significance at either onset or peak by miR-Seq and are regulated in the same direction but at a different time point during EAE by qPCR validation. For these miRNAs, the tag counts of some biological replicates were below 100 counts per million. For qPCR validation, biological replicates are expected to have strong correlation with sequence rates ranging from 10 to 100 000< counts per million^27^. To control for this inherent limitation in NGS, we increased the amount of biological replicates for our qPCR validation. Thus, our qPCR data was considered to be more reliable. From the 27 miRNAs statistically regulated, as identified by qPCR, 10 were downregulated during onset or peak disease; 16 miRNAs were upregulated at onset or peak; and one miRNA, miR-6540-5p, was significantly downregulated at onset and significantly upregulated at peak.

**Table 1:** miR-Seq validated miRNA expression in the EAE neuronal subtypes and as previously described in EAE and/or MS.

miRNA	lumbar motor neurons (2^−ΔΔCt^)				RGC layer (2^−ΔΔCt^)				EAE/MS expression
	onset		peak		peak		chronic		
	FC	p-adj	FC	p-adj	FC	p-adj	FC	p-adj	
miR-novel-chr7_35252	**−91**.**0**	**0**.**0001**	−2.2	0.3856	−1.9	0.1803	1.3	0.5714	N/A
miR-335-5p	**−10**.**9**	**<0**.**0001**	**−4**.**8**	**0**.**0002**	−1.0	0.9619	1.2	0.6167	downregulated in MS NAWM versus HC^21^
miR-novel-chr12_57357	**−4**.**4**	**0**.**0285**	−2.1	0.2195	2.5	0.0708	2.1	0.0970	N/A
miR-novel-chr16_70802	−1.9	0.2347	**−4**.**0**	**0**.**0062**	−1.7	0.1007	1.0	0.9847	N/A
miR-183-5p	**−6**.**0**	**<0**.**0001**	**−4**.**0**	**<0**.**0001**	−1.4	0.6590	1.5	0.3293	*Rag1*−/− mice immunized with MOG_35–55_ and transferred with *miR*-*183*−/− CD4+ T cells show reduced clinical score compared to WT CD4T cells^50^; serum expression showed pathogenic correlation with MS clinical/MRI data^34^
miR-125b-1-3p	−1.2	0.8225	**−3**.**3**	**0**.**0038**	−1.0	0.9966	1.0	0.9896	upregulated in MS NAWM versus HC^21^
miR-129-1-3p	**−3**.**9**	**0**.**0003**	−1.5	0.1016	**−1**.**8**	**0**.**0448**	**−2**.**2**	**0**.**0019**	downregulated in MS NAWM versus HC^21^
miR-92b-5p	−2.0	0.2592	**−3**.**8**	**0**.**0231**	−2.1	0.2233	1.2	0.8287	N/A
miR-127-3p	**−1**.**7**	**0**.**0060**	**−1**.**8**	**0**.**0025**	1.1	0.9071	**−2**.**0**	**0**.**0259**	upregulated in serum exosomes of S/PPMS versus HC^33^; downregulated in female RRMS leukocytes during remission versus HC^39^
miR-148a-3p	**−2**.**2**	**0**.**0003**	−1.3	0.051	**1**.**5**	**0**.**0409**	1.0	0.9002	upregulated in MS inactive lesions versus NAWM^17^; upregulated in T_regs_ from MS patients versus HC^20^; upregulated in PBMCs of RRMS remission versus HC^37^
miR-6540-5p	**−4**.**4**	**0**.**0076**	**4**.**7**	**0**.**0062**	1.7	0.2983	1.1	0.8995	N/A
miR-340-5p	1.3	0.3650	**1**.**9**	**0**.**0346**	**2**.**8**	**0**.**0041**	1.1	0.9369	downregulated in both MS active and inactive lesions versus normal brain WM^17^; upregulated in MS CD4+ T cells versus HC^41^; upregulated in plasma of EAE mice vs control^36^
miR-142a-5p	−1.1	0.8945	**2**.**5**	**0**.**0084**	1.3	0.6656	**2**.**4**	**0**.**0249**	upregulated in MS NAWM versus HC^21^; upregulated in MS NAWM versus HC, and EAE lumbar spinal cord at peak and chronic phases of disease versus control^45^; serum expression showed protective correlation with MS clinical/MRI data^34^
miR-203-3p	−1.0	0.9881	**2**.**8**	**0**.**0241**	**41**.**6**	**<0**.**0001**	1.2	0.6928	upregulated in lymph nodes of EAE-susceptible rats versus non-susceptible controls^29^; downregulated in B cells of RRMS vs HC^43^; upregulated in PBMCs of RRMS remission versus HC, and RRMS remission vs RRMS relapse^37^
miR-490-5p	1.1	0.9755	**2**.**9**	**0**.**0086**	1.3	0.5863	1.1	0.9291	N/A
miR-423-5p	−1.8	0.0959	**4**.**4**	**0**.**0005**	−1.3	0.2228	1.0	0.9863	downregulated in MS CD3+ T cells versus HC^40^
miR-370-5p	−2.3	0.1139	**3**.**9**	**0**.**0122**	1.3	0.6134	**1**.**9**	**0**.**0438**	N/A
miR-205-5p	−1.6	0.6079	**4**.**8**	**0**.**0127**	−1.1	0.9749	**3**.**7**	**0**.**0257**	N/A
miR-1969	−1.7	0.4603	**5**.**5**	**0**.**0044**	5.5	0.0712	**5**.**9**	**0**.**0199**	N/A
miR-7a-5p	2.2	0.4451	**7**.**2**	**0**.**0436**	**3**.**2**	**0**.**0009**	**2**.**1**	**0**.**0037**	downregulated in MS NAWM versus HC^21^; upregulated in lymph nodes of EAE-susceptible rats versus non-susceptible controls^29^; downregulated in CIS/RRMS whole blood versus HC^31^; serum expression showed pathogenic correlation with MS clinical/MRI data^34^
miR-381-3p	−3.8	0.1636	**5**.**7**	**0**.**0324**	**2**.**9**	**0**.**0069**	**2**.**1**	**0**.**0130**	N/A
miR-101a-3p	3.1	0.0971	**8**.**3**	**0**.**0118**	**3**.**3**	**0**.**0006**	1.3	**0**.**2863**	downregulated in RRMS leukocytes versus HC^38^; serum expression showed protective correlation with MS clinical/MRI data^34^
miR-374b-5p	−2.4	0.2454	**8**.**0**	**0**.**0034**	1.1	0.8913	**1**.**6**	**0**.**0069**	N/A
miR-199b-5p	2.5	0.4402	**8**.**6**	**0**.**0247**	1.7	0.0575	**1**.**9**	**0**.**0052**	N/A
miR-novel-chr7_31864	2.1	0.4659	**16**.**8**	**0**.**0032**	1.1	0.9767	1.2	0.8166	N/A
miR-223-3p	**9**.**1**	**0**.**0129**	**19**.**8**	**0**.**0006**	1.3	0.0755	−1.0	0.9778	upregulated in RRMS whole blood versus HC^30^; upregulated in MS active lesions versus NAWM^17^; upregulated in T_regs_ from MS patients versus HC^20^; downregulated in serum of RRMS and PPMS versus HC^32^; upregulated in lymph nodes of EAE-susceptible rats versus non-susceptible controls^29^; upregulated in PBMCs from RRMS vs HC^35^; downregulated in serum from RRMS vs HC^35^; upregulated in MS NAWM vs HC^46^; upregulated in CD4+ T cells during relapse phase of RRMS versus remission and HC^42^; *miR*-*223*−/− mice immunized with MOG_35–55_ show reduced clinical severity versus control^47–49^ upregulated in serum exosomes of RRMS vs S/PPMS, and S/PPMS versus HC^33^; serum expression showed protective correlation with MS clinical/MRI data^34^; upregulated in PBMCs of RRMS remission versus HC^37^
miR-7056-5p	**12**.**6**	**0**.**0111**	**45**.**6**	**0**.**0004**	−1.1	0.9528	**2**.**1**	**0**.**0373**	N/A

miRNAs are organized from most significantly downregulated to most significantly upregulated. Bold FC (fold change) and p-adj (adjusted p-value) values are statistically significant (n = 3–8 RGC layer and n = 3–6 motor neurons, one-way ANOVA, p < 0.05, Dunnett’s multiple comparisons test), N/A = not applicable.

Upregulated miRNAs, in order of increasing expression, included miR-340-5p, miR-142a-5p, miR-203-3p, miR-490-5p, miR-423-5p, miR-370-5p, miR-205-5p, miR-101a-3p, miR-1969, miR-7a-5p, miR-381-3p, miR-374b-5p, miR-199b-5p, miR-223-3p, and miR-7056-5p. Only the two most highly upregulated miRNAs, miR-223-3p and miR-7056-5p, were significantly upregulated at onset and peak. The miRNAs that were downregulated show a more unique biology. miR-novel-chr7_35252, miR-novel-chr12_57357, miR-129-1-3p, and miR-148a-3p were uniquely downregulated at onset whereas miR-novel-chr16_70802, miR-125b-1-3p, and miR-92b-5p were downregulated at peak. miR-335-5p, miR-183-5p, and miR-127-3p were downregulated at both onset and peak EAE.

### Differential miRNA expression over the course of EAE in the RGC layer

**Figure 4:**
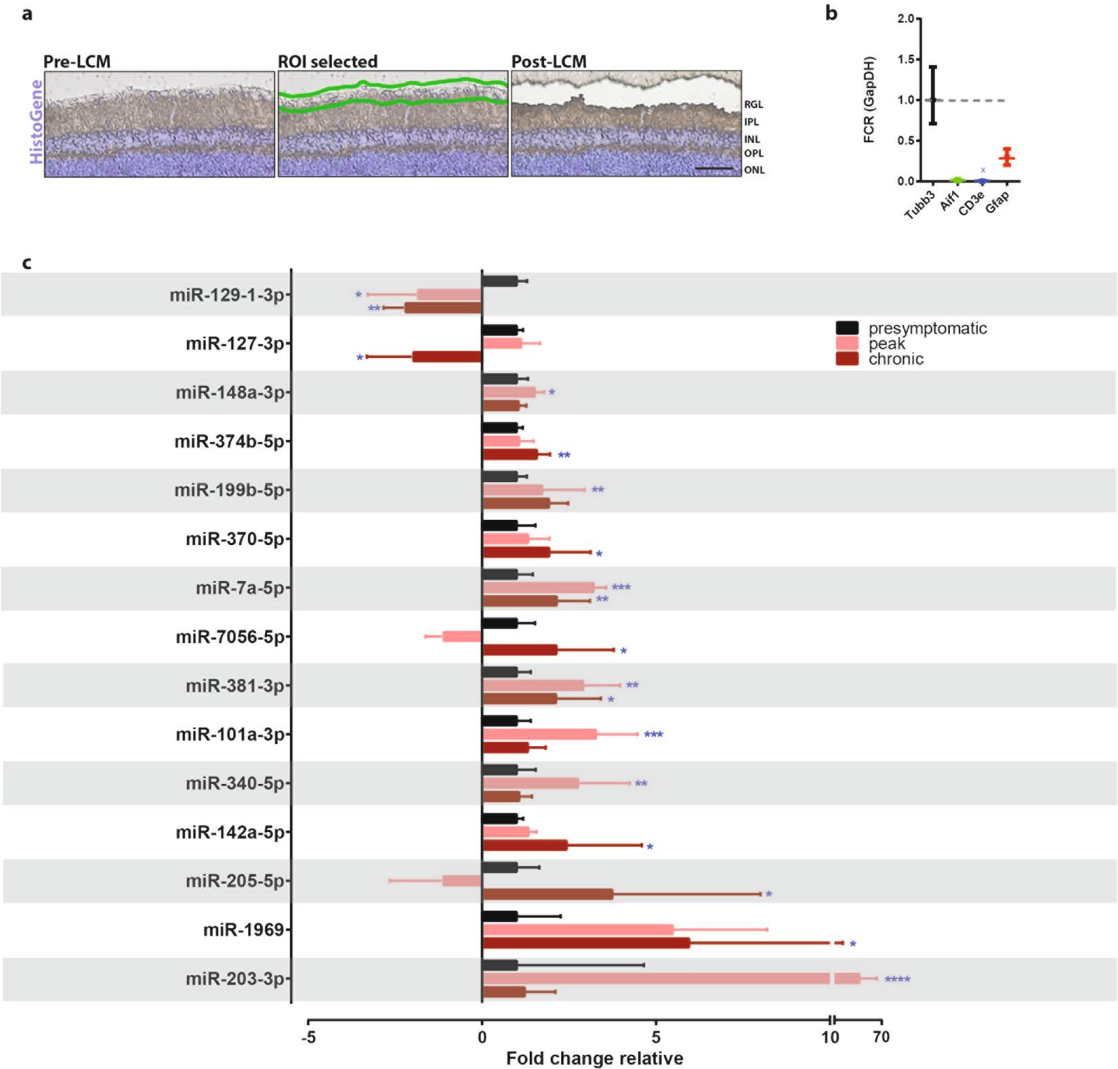
Profiling miRNA expression in the RGC layer of EAE mice. (**a**) Representative retinal sections stained with HistoGene following LCM (ROI = region of interest, RGC = retinal ganglion cell layer, IPL = inner plexiform layer, INL = inner nuclear layer, OPL = outer plexiform layer, ONL = outer nuclear layer), 50 um scale bar. (**b**) qPCR of LCM RGC layer tissue for microglia/macrophages (Aif1), immune cells (Cd3e), and astrocytes (Gfap) relative to neuronal RNA expression (Tubb3)., FCR, Fold Change Range. (**c**) Taqman MicroRNA Assay (qPCR) of miRNAs in the RGC layer of EAE mice over the course of the disease, normalized to endogenous control snoRNA202 for each individual miRNA and depicted as fold change relative to normalized presymptomatic levels. ****p < 0.0001, ***p < 0.001, p** < 0.01, *p < 0.05 (n = 3–8, one-way ANOVA, p < 0.05, Dunnett’s multiple comparisons test).

We next asked if miRNA regulation in lumbar motor neurons was conserved in RGCs, another neuronal population that is affected in EAE and MS. To investigate the miRNA changes in the RGCs, we isolated the RGC layer from EAE mice, which is comprised of approximately 50% RGCs^28^. RGCs were isolated at the presymptomatic stage (before onset of symptoms), peak and chronic stages using LCM (Fig. 4a), and neuronal purity of total RNA was similarly confirmed as in motor neurons (Fig. 4b). Again we saw little or no microglia/macrophages or immune cells, and some astrocyte RNA expression as expected from retinal astrocytes in the RGC layer^28^. We investigated the expression of the 27 miRNAs that were validated in the motor neurons of EAE mice. From these 27 miRNAs, 15 miRNAs were identified as significantly regulated in the RGC layer of EAE mice (Fig. 4c and Table 1); where 14 miRNAs were expressed in the same direction as those in the motor neurons. Specifically, miR-340-5p, miR-101-3p, and miR-203-3p were upregulated at peak; and miR-374b-5p, miR-199b-5p, miR-370-5p, miR-7056-5p, miR-142a-5p, miR-205-5p and miR-1969 were upregulated at chronic EAE. At both peak and chronic stage, miR-381-3p and miR-7a-5p were upregulated. Interestingly, miR-148a-3p was upregulated in the RGC layer, while it was downregulated in the motor neurons. From the downregulated miRNAs in motor neurons, only miR-129-1-3p and miR-127-3p were also downregulated in the RGC layer. None of the novel miRNAs identified in the motor neurons remained significantly regulated in the RGC layer. This gave approximately 50% overlap in miRNA expression profiles between the two neuronal subtypes. These results are summarized in Fig. 4 and Table 1 for the lumbar motor neurons and the RGC layer of EAE mice.

### miR-Seq identified and validated miRNAs in EAE neurons are found regulated in other EAE/MS tissue

Next, we asked if the miRNAs identified by miR-Seq and validated in the neurons of EAE mice were previously described as differentially regulated in other tissues and cells in EAE and/or MS. Of the 27 regulated miRNAs in motor neurons, 13 have been previously implicated in EAE and/or MS (Table 1). Regulation of individual miRNAs was previously identified in immune populations including lymph nodes^29^, whole blood^30,31^, serum^32–35^, plasma^36^, peripheral blood mononuclear cells (PBMCs)^35,37^, leukocytes^38,39^, CD3+ T cells^40^, CD4+ T cells^41,42^, T regulatory cells (T_regs_)^20^, or B cells^43^; or in the brain within MS lesions^17,44^ or normal appearing white matter (NAWM)^21,45,46^. In a small number of cases the miRNA of interest was genetically manipulated for investigation in EAE^47–50^. The miRNAs overlapping with those previously implicated in EAE and/or MS include miR-101-3p, miR-125b-1-3p, miR-127-3p, miR-129-1-3p, miR-142a-5p, 148a-3p, miR-183-5p, miR-203-3p, miR-223-3p, miR-335-5p, miR-340-5p, miR-423-5p, and miR-7a-5p. Intriguingly the only miRNAs that were regulated in the same direction in neurons as in previously described tissue were miR-129-1-3p, miR-142a-5p and miR-335-5p, all regulated in MS NAWM^21^.

### *In silico* assessment of putative targets identifies novel pathways predicted to be affected in the neurons of EAE mice

**Figure 5:**
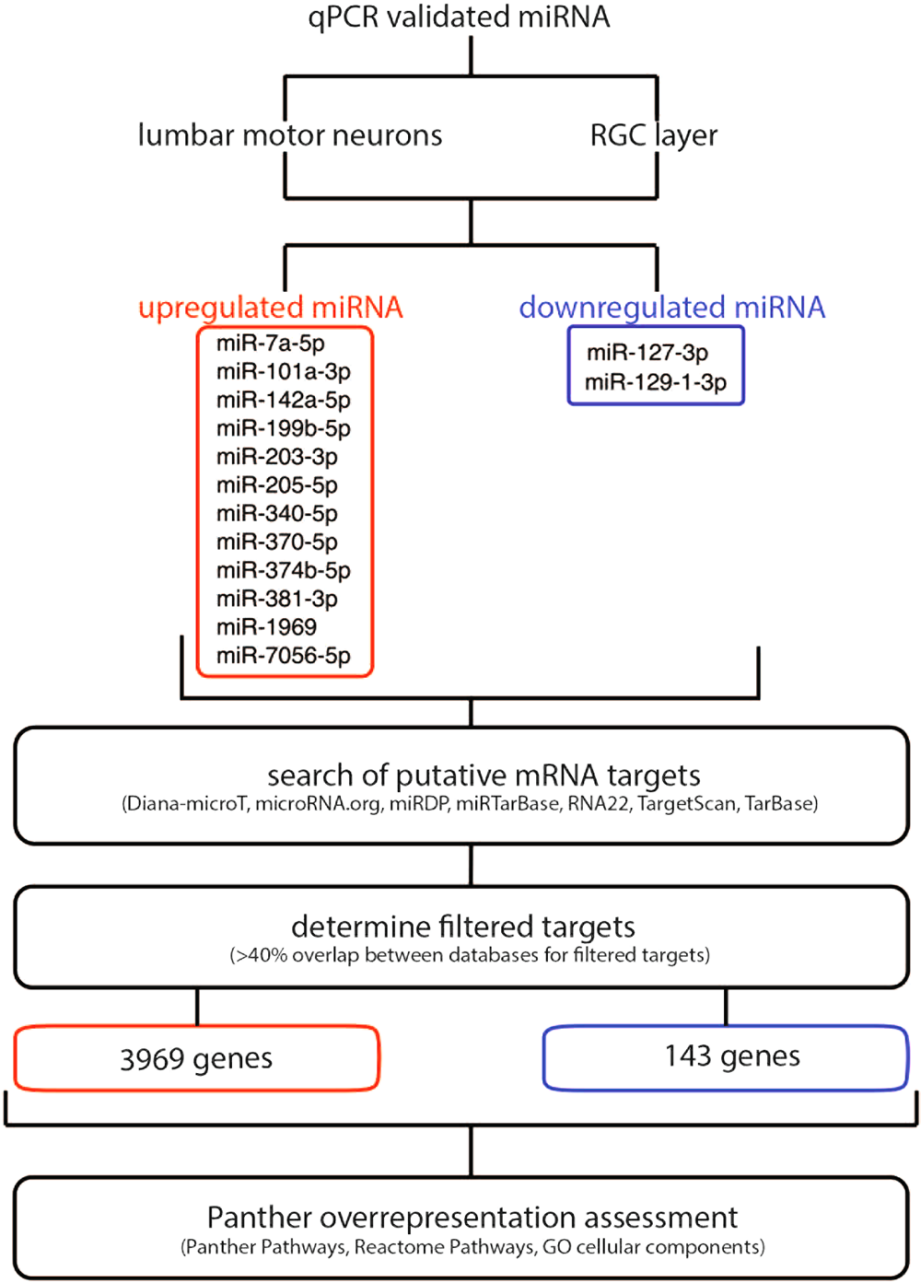
Flow-through of the *in silico* assessment for putative mRNA targets of the differentially regulated miRNAs in the lumbar motor neurons and RGC layer of EAE mice.

To predict programs of gene expression that may be regulated by miRNAs that were commonly regulated in motor neurons and RGCs, we performed a non-biased *in silico* analysis to identify putative mRNA targets of regulated miRNAs (Fig. 5).

**Figure 6:**
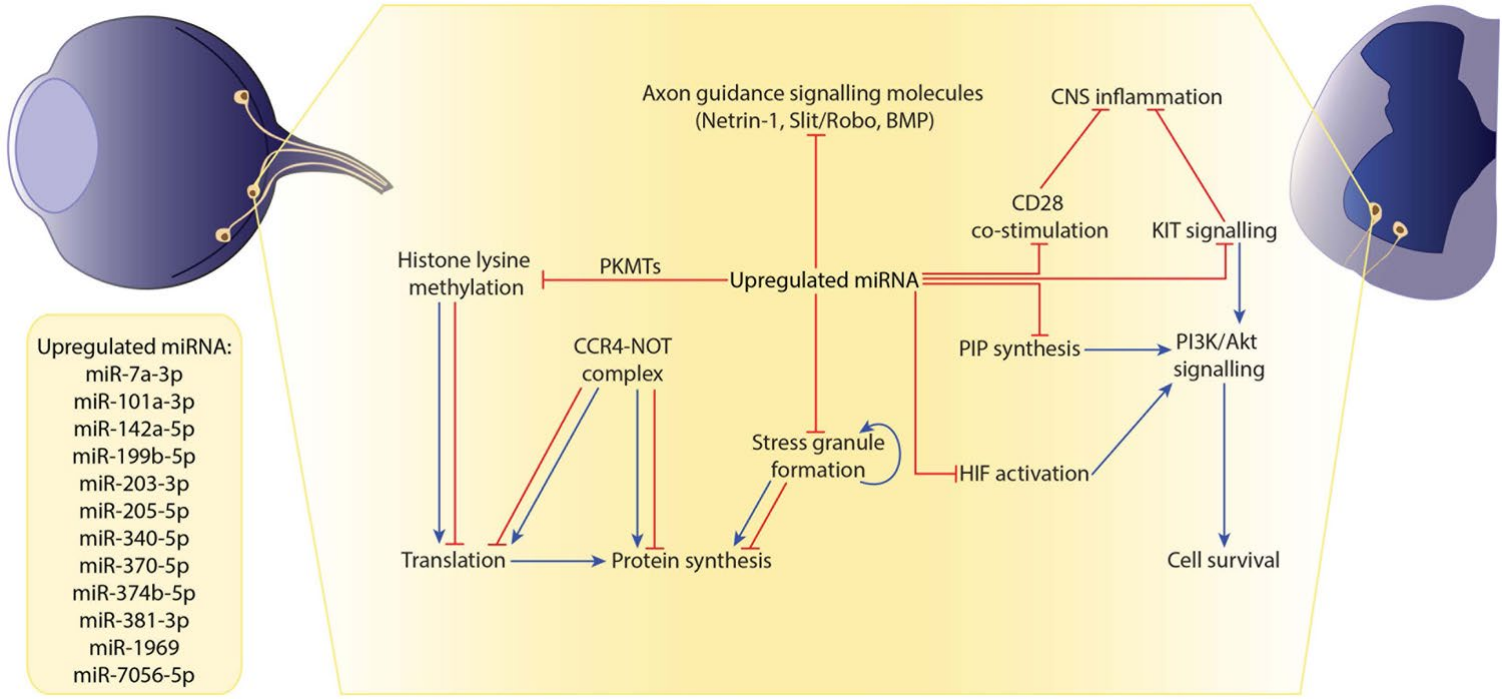
The upregulated miRNAs of neurons during EAE potentially block neuroprotective responses to inflammation. Upregulated miRNAs block axon guidance cues, PIP synthesis, CD28 co-stimulation, HIF activation, KIT signaling, and transcriptional and post-transcriptional regulation pathways, as determined by an *in silico* approach. PI3K/Akt signaling for potential cell survival is blocked by the inhibition of PIP synthesis, HIF activation and KIT signaling. Inhibition of KIT signaling, along with CD28 co-stimulation, prevents any potential dampening of CNS inflammation. Inhibition of several axon guidance cues occurs via the inhibition of signaling through Netrin-1, Slit/Robo and BMP. Finally, inhibition of PKMT histone lysine methylation, CCR4-NOT complex assembly, and stress granule formation converge on many levels of transcriptional and post-transcriptional regulation.

We mined predicted targets of miRNAs using Diana-microT^51,52^, microRNA.org^53^, miRDP^54^, miRTarBase^55^, RNA22^56^, TargetScan^57^, and TarBase^58^. mRNAs that were predicted targets in at least 3 of 7 databases were coined ‘filtered targets’. To determine conserved pathways between the neuronal subtypes in EAE, we overlapped the filtered targets of regulated miRNAs in lumbar motor neurons and the RGC layer and performed an overrepresentation test using Protein ANalysis THrough Evolutionary Relationships (PANTHER) tools^59,60^. We identified several enriched PANTHER Pathways, Reactome Pathways and gene ontology (GO) cellular components that are targeted by upregulated miRNAs (Table 2). Specific gene targets identified in the PANTHER, Reactome and GO cellular component pathways are recorded in Supplementary Tables S3–5, respectively. The upregulated miRNAs in neurons harvested from EAE mice are predicted to target and downregulate genes in PANTHER Pathways including hypoxia response via hypoxia-inducible factors (HIF) activation, and axon guidance mediated by Slit/Robo. Predicted Reactome Pathways include: KIT signaling; signaling by bone morphogenic protein (BMP); netrin-1 signaling; CD28 co-stimulation; protein lysine methyltransferases (PKMTs) methylate histone lysines; and synthesis of phosphatidylinositol phosphates (PIPs) at the plasma membrane. PANTHER and Reactome analyses differ in how they classify terms^59^. The GO cellular components targeted by the upregulated miRNAs are the CCR4-NOT complex, and cytoplasmic stress granules (SG). The predicted targets suggest that upregulated miRNAs target pathways converging on cell survival and growth, cytoskeleton rearrangement, and the stress response. These pathways and how they may act in concert are summarized in Fig. 6.

**Table 2:** Overrepresented pathways and cellular components by the upregulated miRNAs in EAE neurons.

	Fold enrichment	p-value
**Panther Pathways**		
Hypoxia response via HIF activation	3.32	0.00416
Axon guidance mediated by Slit/Robo	3.30	0.02160
**Reactome Pathways**		
Regulation of KIT signaling	4.53	0.03230
Signaling by BMP	3.69	0.03460
Netrin-1 signaling	3.69	0.03460
CD28 co-stimulation	3.51	0.01080
PKMTs methylate histone lysines	3.24	0.01150
Synthesis of PIPs at the plasma membrane	3.16	0.02580
**GO Cellular Component**		
CCR4-NOT complex	4.16	0.01130
cytoplasmic stress granule	3.10	0.00442

**Figure 7:**
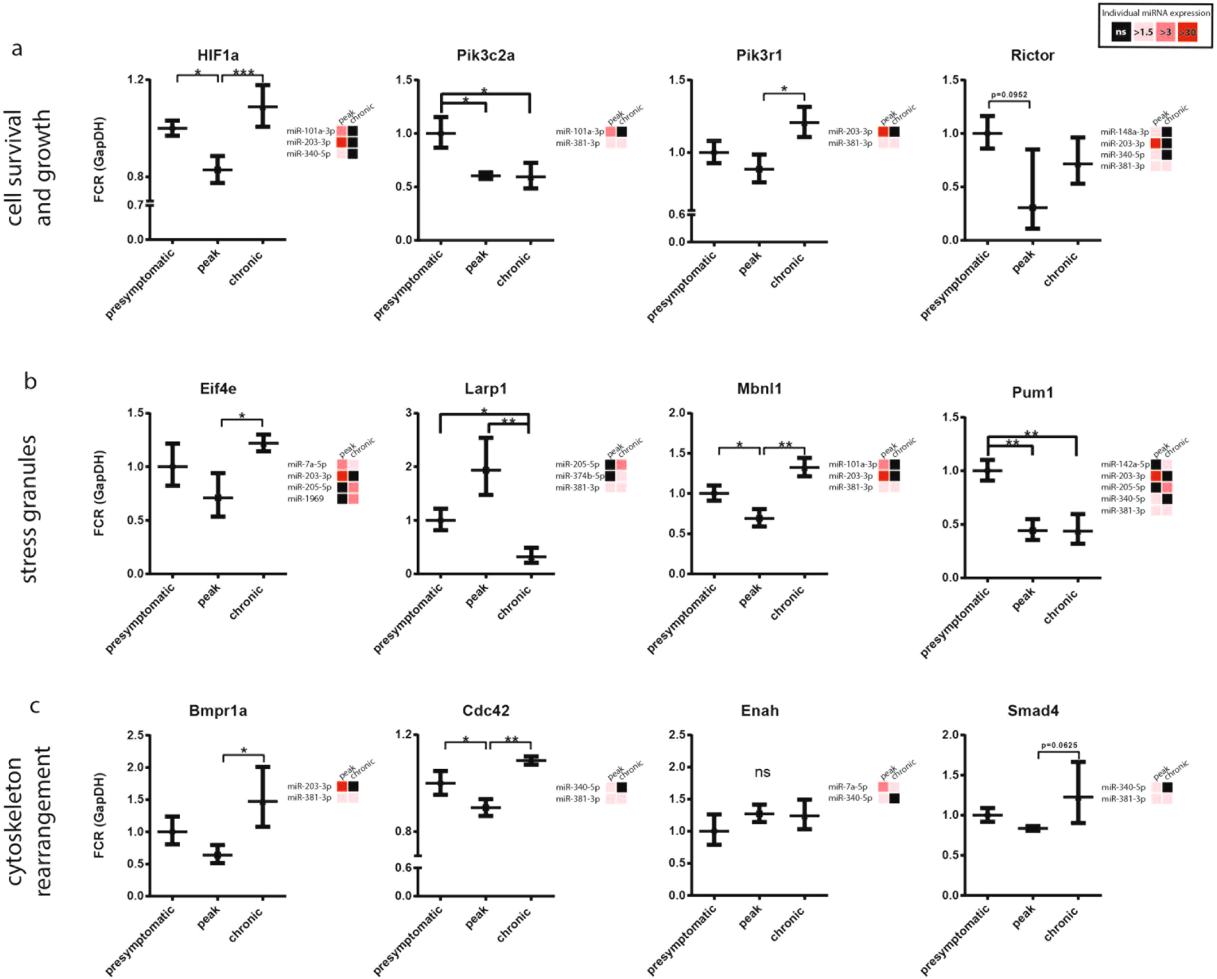
Expression of predicted miRNA gene targets as identified by *in silico* analysis in the RGC layer. Right-hand side of every qPCR profile for target genes is their respective targeting miRNA expression profiles with fold upregulation relative to presymptomatic levels. Black, not significant (ns). Genes are organized by their physiological roles, specifically (**a**) cell survival and growth (**b**) stress granule formation, and (**c**) cytoskeleton rearrangement. Gene expression was normalized to endogenous GapDH, FCR = Fold Change Range. ***p < 0.001, **p < 0.01, *p < 0.05 (n = 3–6, one-way ANOVA, p < 0.05, Tukey’s multiple comparisons test).

To validate if these *in silico* identified pathways are regulated, we evaluated the expression of key genes described in Supplementary Tables 3–5, focusing on representative genes regulating cell survival and growth, cytoskeleton rearrangement, and the stress response; as well as genes targeted by multiple miRNAs. Gene expression was assessed in the RGC layer over the EAE disease course, and compared to the miRNA expression of the respective targeting miRNAs (Fig. 7). Genes *Bmpr1a*, *Cdc42*, *Hif1a*, *Pik3r1*, *Eif4e*, and *Mbnl1* showed a significant downregulation at peak disease, corresponding with increased expression of their targeting miRNAs at peak disease. Genes *Rictor* and *Smad4* showed the same trend, but were not significant. *Larp1* was uniquely downregulated at chronic disease stage, and accordingly its targeting miRNAs were upregulated at the chronic stage. Genes *Pik3c2a* and *Pum1* were downregulated throughout EAE relative to presymptomatic levels. *Pum1* is targeted by the most miRNAs, and showed the strongest downregulation throughout EAE, emphasizing the validity of our *in silico* approach for determining the (1) filtered targets of our differentially regulated miRNAs; and (2) PANTHER pathways and cellular components assessment of the filtered targets. The overrepresentation test for filtered targets of miRNAs that were downregulated in both neuronal subtypes generated a small list of 143 genes, and this was insufficient to identify overrepresented pathways or cellular components. Therefore, we performed an overrepresentation analysis for pathways that were predicted to be downregulated in lumbar motor neurons alone. We identified several enriched PANTHER Pathways, Reactome Pathways, and GO cellular components that were targeted by downregulated miRNAs (Table 3). The PANTHER Pathways included signaling mediated by the following: histamine H_1_ receptor; oxytocin receptor; phosphoinositide 3-kinase (PI3K); thyrotropin-releasing hormone receptor; Alzheimer’s disease (AD)-amyloid secretase; epidermal growth factor receptor (EGFR); 5-HT_2_ type receptor; and fibroblast growth factor (FGF). The Reactome Pathways included: post-transcriptional silencing by small RNAs; signaling by TGF-β Receptor Complex; and several events activating PI3K/Akt via GAB1 signalsome, EGFR, and FGF receptor (FGFR). Finally, the cellular components enriched in targeted genes of downregulated motor neuron miRNAs included protein phosphatase type 2A (PP2A) complex, and endocytic vesicles. Specific gene targets identified in the PANTHER Pathways, Reactome Pathways, and GO cellular components are recorded in Supplementary Tables S6–8, respectively. As these pathways and cellular components were targeted by the downregulated miRNAs, it is suggested that they are now activated or relieved of inhibition.

**Table 3:** Overrepresented pathways and cellular components by the downregulated miRNAs in EAE lumbar motor neurons.

	Fold enrichment	p-value
**Panther Pathways**		
Histamine H_1_ receptor mediated signaling pathway	3.77	0.03600
Oxytocin receptor mediated signaling pathway	3.68	0.00696
PI3 kinase pathway	3.57	0.03120
Thyrotropin-releasing hormone receptor signaling pathway	3.50	0.01160
AD-amyloid secretase pathway	3.34	0.01870
EGF receptor signaling pathway	3.34	0.00001
5-HT_2_ type receptor mediated signaling pathway	3.20	0.02930
FGF signaling pathway	3.15	0.00024
**Reactome Pathways**		
Post-transcriptional silencing by small RNAs	13.51	0.01070
Signaling by TGF-beta Receptor Complex	3.94	0.00829
GAB1 Signalsome	3.15	0.00970
PI3K events (ERBB4, FGFR4, FGFR3, FGFR2, FGFR1)	3.09	0.02100
PIP3 activates Akt signaling	3.09	0.02100
PI3K/Akt activation	3.00	0.03150
**GO Cellular Component**		
PP2A complex	6.64	0.04940
endocytic vesicle	3.60	0.00001

## Discussion

In this study, we took an unbiased approach to determine the changes in neuronal miRNA expression in a model of CNS inflammation. We compared the miR-Seq validated profile of lumbar motor neurons to RGCs in EAE, and identified potentially regulated pathways and cellular components during neuroinflammation, using an *in silico* bioinformatics approach. This provides the first analysis of miRNA expression in neurons over the course of EAE. Currently only immune-related material^20,29–33,35,61^; or CNS tissue with mixed cell types such as lesions^17^ or NAWM^21,46^ have been isolated to profile differential miRNA expression in MS.

The time course assessment of miRNA expression revealed massive upregulation at peak disease. The loss of motor neurons and axons in the ventrolateral and dorsal columns begins early at onset of EAE disease; thus, we hypothesized that lumbar motor neurons would exhibit miRNA regulation at disease onset^11,62^. Rather, almost all miRNAs were uniquely upregulated at peak disease alone, in some cases trending towards upregulation at onset (Fig. 3). Animals may have slightly different kinetics of disease and have more variability at onset.

There are several potential explanations for exaggerated miRNA upregulation at peak disease. Specifically, miRNAs may be trapped in the cell body compartment as result of axonal transection and compromised axonal transport early in EAE, leading to their accumulation over the course of disease^9,62^. Alternatively, it is predicted that genes involved in post-transcriptional silencing by small RNAs would be dramatically enriched 13.5 fold as a consequence of downregulation of select miRNAs in motor neurons (Table 3). The predicted increase in neuronal RISC pathway components contrasts with a report of decreased miR-mediated transcriptional silencing in the T cells and oligodendrocytes of EAE mice^23^. Lastly, it is also possible that negative feedback loops required to limit miRNA expression may be compromised in inflamed neurons. For example, miR-223-3p is part of an autoregulatory negative feedback loop with transcription factor E2F1, and disruption of this loop may contribute to the progression of acute myeloid leukemia^63^. Dysregulation of such feedback loops may thus be affected in the neurons of EAE mice.

Next, our comparison of miRNA expression across cell types in EAE and/or MS revealed that miRNA expression is dependent on the cell type even when compared within the same disease model or group of patients (Table 1). Of the differentially expressed miRNAs in motor neurons, half were regulated in the same direction in the RGC layer. This identifies some similarities in neuronal responses to inflammation irrespective of neuronal function. Differences in miRNA regulation in the two neuronal populations could represent physiologic diversity in responses. Alternatively, these divergences could reflect differences in the time course of inflammation in the motor and visual system. Motor neuron loss begins as soon as 14 dpi in the EAE model^11^, whereas RGC loss becomes significant between 25 and 40 dpi^11,13^. Another consideration is that motor neurons and their axons can be exposed to a common inflammatory milieu whereas RGCs are compartmentalized from their axons within the optic nerve. Similar proportions of T cell, B cell, macrophage and microglia populations have been described within the spinal cord and optic nerve^64^. However, there is a lack of immune infiltrates within the choroid or retina surrounding the RGCs, with CD3+ infiltrates exclusively occurring in the optic nerve whereas activated microglia and astrocytes are present in the choroid^12,65^. Alternatively, in the spinal cord, CD3+ infiltrates are described in close proximity to motor neurons^11^. We cannot rule out the possibility that some differences result from comparing pure motor neurons to the RGC layer, which contains amacrine cells and retinal astrocytes in addition to approximately 50% RGCs^28^.

When comparing our analysis of neurons to prior studies examining EAE or MS tissue, we find that most of the neuronally-regulated miRNAs were not similarly regulated in other EAE/MS tissue. Several miRNAs including miR-127-3p, miR-223-3p, miR-7a-5p, miR-203-3p and miR-340-5p are differentially regulated depending on the tissue source (Table 1). For example, miR-340-5p was reportedly upregulated in CD4+ T cells in MS but downregulated in MS lesions^17,41^. The only prior study to investigate neuronal miRNAs compared miRNA expression by fluorescence intensity between myelinated and demyelinated hippocampal human post-mortem MS tissue^44^. They investigated a list of candidate miRNAs based on their specific associations with mRNAs that are changed in demyelinated MS hippocampi. Regulated miRNAs in that study were not differentially regulated in our screen.

Our results highlight the importance of performing cell type specific analyses of miRNAs because target genes can have distinct effects in individual cell types. For example, miR-183-5p is elevated in the neurons of a model of spinal muscular atrophy (SMA) and promotes neurodegeneration^66^. However, miR-183 elevation is also important for blocking the cytotoxic effects of natural killer (NK) cells by targeting required receptors for NK cell activation^67^. This could have potential protective roles in the inflammatory context of MS^68^. In both models miR-183-5p is upregulated, yet its elevation produces drastically different biologies in the two cell types.

Finally, the putative pathways targeted by the differentially regulated neuronal miRNAs suggest that these miRNAs contribute to axonal pathology and cell death. Using a bioinformatics approach, we determined putative pathways targeted by upregulated miRNAs during neuroinflammation. Many target pathways such as CD28 co-stimulation are known to affect the biology of non-neuronal cells (Supplementary Tables S3 and 4). While it is possible that neuronal miRNAs may be released in exosomes to affect the biology of other cells^33^, we will limit our discussion to potential roles in the neuronal response.

Many of the pathways and cellular components described in Table 2 converge on cell survival and growth, cytoskeleton dynamics, and stress response. The predicted direction of regulation suggest that regulated miRNAs could contribute to neuronal degeneration, cell death and an aberrant stress response.

Many genes of the pathways described in Table 2 converge on the promotion of cell survival and growth via the PI3K/Akt/mTOR cascade^69,70^. Genes *Src*, *Mtor*, *Rictor*, *Rptor*, *Akt1*, *Akt2*, *Akt3*, *Pik3r1*, and *Pik3c2a* are directly involved in positive mTOR signaling^70,71^, and are predicted to be targeted by several of our upregulated miRNAs (Supplementary Table S4). Such a regulation would result in stunted growth and cell death. HIF signaling, downstream of mTOR, promotes survival during hypoxia^70,72^. HIF signaling genes *HIF1a* and *Arnt* are also targeted by our upregulated miRNAs^72^. *In vivo* knockdown or inhibition of HIF activity limits axon regeneration in axotomized sensory neurons^73,74^, and exacerbates cerebral ischemia-induced tissue damage^75^; whilst the pharmacological upregulation of HIF in animal models of cerebral ischemia^76^, Parkinson’s disease^77^, and AD^78^ alleviates disease. Targeting of the PI3K/Akt/mTOR cascade and its downstream pathways suggests our upregulated miRNAs promote cell death of EAE neurons. This is emphasized by the significant downregulation of genes *HIF1a*, *Pik3c2a* and *Pik3r1* at peak disease in the RGC layer of EAE mice (Fig. 7a).

Another pattern that emerged was that stress granule (SG) formation may be disrupted in response to miRNA upregulation (Tables 2 and S5). Many SG initiating genes, including *Tia1*, *Tial1*, *G3bp1*, *Zfp36*, *Fmr1*, *Pum1*, and *Pum2*, are targeted by the upregulated miRNAs^79–83^, along with other essential components of SGs listed in Supplementary Table S5. Some of these genes were downregulated throughout EAE, with *Pum1* significantly downregulated at both peak and chronic EAE in the RGC layer; where *Pum1* was one of the most targeted genes by the upregulated miRNAs (Fig. 7b). SGs are non-membrane bound cytoplasmic aggregates of RNA and protein that form in response to environmental stress such as hypoxia, endoplasmic reticulum (ER) stress, reactive oxygen species, and nutrient deprivation^84^. ER stress is a hallmark of MS and chronic stress in neurons that leads to failed SG assembly^85–87^. SGs are transient and can act to protect untranslated mRNA from stress-dependent damage^88^. Use of the SG-stabilizing drug guanabenz in EAE alleviates clinical symptoms^89,90^. The implication from our study is that upregulated miRNAs may block adaptive SG formation, limiting the ability of neurons to recover from chronic inflammatory stress associated with EAE.

Interestingly, we also identified genes ascribed to axon guidance pathways as targets of upregulated miRNAs. *Mena*, *Rac1*, *Cdc42*, *DCC*, *Smad4*, and *Bmpr1a* are some predicted gene targets of the upregulated miRNAs (Supplementary Tables S3 and 4). Several of these targets can positively influence cytoskeleton rearrangement, such as *Bmpr1a* and *Cdc42*, which we identified as significantly downregulated at peak disease in the RGC layer (Fig. 7c). This raises the possibility that miRNA-dependent downregulation contributes to the formation of retraction bulbs in MS lesions and failure to reform a new growth cone and mount a regenerative response^91–95^.

The current understanding of how neurons are affected during EAE and MS has been unclear. Our assessment of the affected miRNAs and their targeted pathways over the course of EAE suggests that the upregulated miRNAs themselves target pathways related to cell survival and growth, cytoskeletal rearrangement, and stress response. We also identified the downregulation of representative targets in the same tissue, validating our *in silico* approach and emphasizing the contribution of these pathways to EAE. This novel information concerning the neuronal response lends information on what we may be able to target therapeutically to promote neuroprotection or repair for a disease largely discussed in an inflammatory context alone.

## Methods

### Active EAE Disease Induction and Scoring

EAE was induced in 6–9 week old female C57BL/6 mice as previously published^96^. Animals were immunized subcutaneously with 200 μg of myelin oligodendrocyte glycoprotein 35–55 (MOG_35–55_) (MEVGWYRSPFSRVVHLYRNGK; Alpha Diagnostic International) in a 100 μl emulsion of Complete Freund’s Adjuvant (4 mg/ml *Mycobacterium tuberculosis*; Fisher Scientific). On day 0 and day 2, Pertussis toxin (500 ng PTX, Sigma-Aldrich) was injected intra-peritoneally (i.p.). Animals were scored as follows: 0 = normal; 1 = limp tail; 2 = slow righting-reflex; 2.5 = difficulty walking/ataxia; 3 = paralysis of one hindlimb (monoparalysis); 3.5 = hindlimb monoparalysis and severe weakness in the other hindlimb; 4 = paralysis of both hindlimbs (paraparalysis); 4.5 = hindlimbs paraparalysis and forelimbs weakness; 5 = moribund (requires sacrifice). All animal procedures were approved by the Centre de Recherche du Centre Hospitalier de l’Université de Montréal Animal Care Committee (N11023APs) and followed guidelines of the Canadian Council on Animal Care.

### CNS tissue isolation

Animals were injected with a lethal dose of Euthanyl® (Pentobarbital Sodium). Animals showing no sign of pedal and palpebral reflex were perfused intracardially using cold saline. The spinal cord and retina were isolated and frozen at −80 °C in Tissue-Tek® O.C.T. compound. Spinal cords were collected at naïve (score 0), onset (score 0.5–1), and peak (score 3–3.5) clinical points; and retinae were collected at presymptomatic (score 0, 8 dpi), peak (score 3.5–4), and chronic endpoints (score 2, endpoint at 35 dpi). Frozen sections were obtained using a Leica® cryostat CM3050S. Sections were mounted onto RNase-free MembraneSlide 1.0 polyethylene-napthalate (PEN) (Zeiss), at a thickness of 10 μm. Slides were stored at −80 °C until further processing by LCM.

### Laser capture microdissection

Individual MembraneSlide 1.0 PEN were fixed, stained, washed and dried using the Arcturus® HistoGene® LCM Frozen Section Staining Kit (ThermoFisher) following manufacturer’s recommendations. Individual slides were thawed and dried for 20 sec on an RNAase-free glass surface; fixed in 70% ethanol for 30 sec; washed in water for 30 sec; stained with HistoGene for 30 sec; washed in 70% ethanol for 30 sec; washed in 95% ethanol for 30 sec; washed in 100% ethanol for 30 sec; dehydrated in xylene for 5 min; and dried to remove excess xylene for 1 min. All solutions and materials used were RNase-free. HistoGene-stained large motor neuron cell bodies from the ventral horn, and RGC layers from the retina, were laser dissected by PALM MicroBeam (Zeiss) and collected in 15 μl Qizol (QIAGEN) into the cap of an RNase-free 200 μl centrifuge tube. Collected material was vortexed and left at room temperature (RT) for 1 min before being stored at −80 °C until further RNA extraction. Overall, approximately 3000 motor neurons and 700 RGC layers were captured per animal by combining multiple serial sections.

### RNA extraction from LCM tissue

Material from each frozen 200 μl centrifuge tube were pooled together for each individual mouse for RNA extraction. Collected material was topped off to 700 μl QIAzol, and 140 μl of chloroform. Samples were shaken, incubated at RT for 5 min and centrifuged (12, 000 g at 4 °C for 15 min). The upper phase was mixed with 2 times volume of 100% ethanol and 1.5 μl GlycoBlue™ Coprecipitant (ThermoFischer), and left at −80 °C overnight to precipitate all nucleic acids. Precipitant was centrifuged at 16,000 g at 4 °C for 30 min. Liquid was removed and the pellet containing the nucleic acid was washed two times with 400 μl ice cold 75% EtOH. The pellet was air dried for 10 min, resuspended in 10 μl water, and suspended in 700 μl QIAzol. RNA was extracted from the resuspended pellet using the miRNeasy kit (QIAGEN) as per manufacturer’s instructions.

### Cell cultures

All studies were approved by the McGill University Animal Care and Use Committee. To obtain astrocyte and microglial cultures, cortices were dissected from P2-P4 CD1 mouse pups in cold Lebovitz L15 medium (ThermoFisher), and seeded onto dishes coated with 100 μg/mL poly-L-lysine (PLL). At approximately 11 days *in vitro* (DIV), T75 flasks containing a mixed cortical culture were shook for 30 min at 180 rpm on an orbital shaker to remove microglia. Supernatant containing microglia was spun down at 1 rpm for 5 min to pellet cells, pelleted cells were suspended in QIAzol. Simultaneously fresh DMEM 10% fetal bovine serum (FBS) containing 1% penicillin-streptomycin and 2 mM glutamine were added to the cultures. The flasks were left at 37 °C and 5% CO_2_ for 1 h and later shook at 240 rpm for 2 h in order to remove oligodendrocyte precursor cells. Astrocytes were lysed with QIAzol. To obtain cortical neurons, cortices were dissected from embryonic day 16–17 (E16–17) C57/Bl6 mice and dissociated with 0.25% EDTA-trypsin for 20 min at 37 °C, 5% CO_2_, followed by mechanical tituration 10 times with a sterile 1000 uL pipette tip in DMEM 10% FBS containing 1% penicillin-streptomycin and 2 mM glutamine, and seeded onto PLL-coated dishes. Media was changed to Neurobasal medium supplemented with 2% B27, 1% N2, 1% penicillin-streptomycin and 2 mM glutamine 1 h after seeding. At 5 DIV, medium was removed and neurons lysed with QIAzol. To obtain PBMCs, whole blood was collected by cardiac puncture from adult C57Bl/6 mice. Mouse PBMCs were separated by Ficoll-Plaque density centrifugation, and suspended in QIAzol. RNA was extracted from QIAzol lysed cells using the miRNeasy kit (QIAGEN) as per manufacturer’s instructions.

### Next-Generation Sequencing (NGS)

Total extracted RNA from the lumbar motor neurons was sent to Arraystar, Inc for NGS. Total RNA was isolated from each sample and used to prepare the miRNA sequencing library with the following steps: (1) 3′-adapter ligation; (2) 5′-adapter ligation; (3) cDNA synthesis; (4) PCR amplification; (5) size selection of approximately 130–150 bp PCR amplified fragments (corresponding to approximately 15–35 nt small RNAs). The libraries were denatured as single-stranded DNA molecules, captured on Illumina flow cells, amplified *in situ* as clusters and finally sequenced for 51 cycles on Illumina HiSeq2000 according to manufacturer’s instructions. The sequencing reads were quality controlled by CHASTITY onboard the sequencing system with Q-scores. The FASTQ files were processed by a combination of FastQC v.0.11.2, SolexaQA v.2.0, fastq_screen_v0.4.4 (−subset 1500000), and Perl 10 script to remove the reads that contained ambiguous bases “N”, poly(A/T); and the read lengths <14. Q-score fols were 25 for the 3′-end, 10 for <4 bases or 13 for <6 bases in the first 30 bases, and 20 for >75% of all bases. The adaptor sequences were trimmed with cutadapt called from Python environment with–overlap set to 2. Trimmed reads were aligned to a combined mouse pre-miRNA in miRBase 21 and the predicted novel pre-miRNAs with Novoalign v2.07.11, allowing no more than 1 mismatch. miRDeep2 was used to predict novel miRNAs with the trimmed reads. miRNA read counts were processed and normalized with the DeSeq2 (version 3.5) Bioconductor package and used to detect differentially expressed miRNAs in lumbar motor neurons^97^. Significantly differentially expressed miRNAs (adjusted p < 0.05) were visualized as heatmaps created with the Morpheus tool (http://software.broadinstitute.org/morpheus/). *Quantitative RT*-*PCR* (*qRT*-*PCR*) - For qRT-PCR validation of miRNA expression in lumbar motor neurons, samples from deep sequencing and additional naïve and peak time points samples were analyzed (n = 3–6). The expression of 41 differentially regulated miRNAs was analyzed using multiplex qRT-PCR. Forty-one miRNAs were profiled using a Taqman MicroRNA Assay, of which 5 were novel miRNAs that required custom designs (Supplementary Table S2). Multiplexed RT reactions were performed using a mix of miRNA-specific RT primers and a TaqMan miRNA RT kit, with 10 specific primers per RT reaction and 10 ng of RNA per RT reaction. A pre-amplification reaction was run with 5 μl of the RT-generated cDNA using again a pooled set of TaqMan miRNA-specific probes to increase the number of target copies, as previously described^98^. Individual miRNA expression assays were performed using specific miRNA TaqMan probes on the pre-amplified cDNA material. Small nuclear RNA (snRNA) snoRNA202 was used as an endogenous control. Fold change calculations for miRNA expression were performed using the 2^−ΔΔCt^ method^25^, with normalized miRNA expression compared to the naïve mouse controls (score 0). miRNAs identified as significantly regulated in the lumbar motor neurons were assessed in the RGC layer of EAE mice using the multiplex qRT-PCR system with miRNA expression compared to presymptomatic mouse controls (score 0). For the RGC layer, 3–8 animals were assessed per condition. Statistical analysis was done using one-way ANOVA, and a Dunnett’s multiple comparisons test (GraphPad Prism 6). For qRT-PCR of mRNA, total RNA from cell culture or LCM tissue was transcribed using Superscript Vilo or IV Vilo cDNA Synthesis Kit (ThermoFisher), respectively, using manufacturer’s instructions. Individual gene expression was determined using FAM-labeled Taqman probes, and as endogenous controls FAM-labeled GapDH probe for cell culture tissue and VIC-labeled GapDH probe for LCM tissue. For determination of neuronal purity in LCM tissue, RNA markers for other cell types were relative to neuronal Tubb3 expression (n = 3) after GapDH normalization. For assessment of *in silico* identified genes in LCM RGC layer tissue, mRNA expression was compared to presymptomatic levels. Statistical analysis was done using one-way ANOVA, and a Tukey’s multiple comparisons test with n = 3–6 per disease stage (GraphPad Prism 6).

### *In silico* assessment of predicated targets

For significantly regulated miRNAs, a bioinformatics assessment of putative target genes was performed based on a comparative analysis by seven prediction programs. These include: Diana-microT^51,52^, microRNA.org^53^, miRDP^54^, miRTarBase^55^, RNA22^56^, TargetScan^57^, and TarBase^58^. Only mRNAs that were identified as putative targets across 3 of the 7 prediction programs were analyzed further. These were termed as “filtered targets”. To determine conserved pathways between the neuronal subtypes in EAE, we overlapped the filtered targets of the upregulated miRNAs in lumbar motor neurons and the RGC layer (3969 genes). The functional classification of the overlapping filtered targets was performed using an overrepresentation test by PANTHER classification system (http://www.pantherdb.org/)^59,60^. The p-values were determined by PANTHER using the binomial statistic with a Bonferroni correction for multiple testing. From the overrepresentation assessment, we focused on PANTHER Pathways, Reactome Pathways and GO cellular components with a Fold Enrichment above 3. An overrepresentation assessment was repeated for the filtered targets of miRNAs downregulated in both neuronal subtypes (143 genes); however, this list was not comprehensive enough to determine any overrepresentation. An overrepresentation test of the filtered targets of the upregulated miRNAs in the motor neurons alone (4346 genes); upregulated miRNAs in the RGC layer alone (4226 genes); downregulated miRNAs in the motor neurons alone (1422 genes) were also performed. Again, the list of filtered targets of downregulated miRNAs in the RGC layer alone (143 genes) was not comprehensive enough to determine any overrepresentation.

## Electronic supplementary material

Supplementary Material
