# Supplementary material for: Neuronal microRNA regulation in Experimental Autoimmune Encephalomyelitis
Source: Sci Rep. 2018 Sep 7;8:13437. doi: 10.1038/s41598-018-31542-y (PMC6128870; doi:10.1038/s41598-018-31542-y)
Supplement: Supplementary file 1 — Supplementary Material [file 41598_2018_31542_MOESM1_ESM.docx]

**Neuronal microRNA regulation in Experimental Autoimmune Encephalomyelitis**

Camille A. Juźwik^1^, Sienna Drake^1^, Marc-André Lécuyer^2^, Radia Marie Johnson^3^, Barbara Morquette^1^, Yang Zhang^1^, Marc Charabati^2^, Selena M. Sagan^4^, Amit Bar-Or^1,5^, Alexandre Prat^2^, Alyson E. Fournier^1*^

1. McGill University, Montréal Neurological Institute, Montréal, QC H3A 2B4, Canada
2. Centre de Recherche du Centre Hospitalier de l'Université de Montréals, Université de Montréal, Montréal, QC H2X 0A9, Canada
3. McGill University, Goodman Cancer Research Centre, Montréal H3A 1A3 Canada
4. McGill University, Departments of Microbiology & Immunology and Biochemistry, Montréal, QC H3G 0B1 Canada
5. Perelman School of Medicine, University of Pennsylvania, Philadelphia, PA 19104 USA

* Corresponding author at: Alyson Fournier, Montréal Neurological Institute, 3801 University Street, room BT-105, Montréal QC H3A 2B4, Canada

Email address: [alyson.fournier@mgill.ca](mailto:alyson.fournier@mgill.ca)

Supplemental Table S1

*Positive controls for qPCR probes of different glial cell and infiltrating immune cell markers*

| **Probe** | **Tissue** | **Ct value** |
| --- | --- | --- |
| Aif1 | microglia (n=1) | 24.503 |
| Aif1 | microglia (n=2) | 24.948 |
| Cd3e | PBMCs (n=1) | 26.363 |
| Cd3e | PBMCs (n=2) | 26.148 |
| Gfap | astrocytes (n=1) | 22.925 |
| Gfap | astrocytes (n=2) | 22.964 |
| Tubb3 | cortical neurons (n=1) | 21.714 |
| Tubb3 | cortical neurons (n=2) | 22.163 |

Supplemental Table S2

*miRNA sequence information for custom design Taqman MicroRNA Assays*

| **miRNA** | **pre-miRNA arm** | **MATURE-SEED** | **MATURE-LENGTH** | **MATURE-SEQ** |
| --- | --- | --- | --- | --- |
| mmu-miR-92b-3p | 3p | AUUGCA | 22 | UAUUGCACUCGUCCCGGCCUCC |
| mmu-miR-novel-chr2_10423 | 5p | GGGCGU | 17 | CGGGCGUGGGGGUGGGG |
| mmu-miR-novel-chr2_7634 | 5p | GGGCUG | 19 | AGGGCUGGAGAGAUGGCUC |
| mmu-miR-novel-chr7_31864 | 5p | CCGAUC | 22 | ACCGAUCCCGGGUUAGUCUCCU |
| mmu-miR-novel-chr7_35252 | 3p | GAUAUA | 22 | UGAUAUAGCCAAGCCCGACUGU |
| mmu-miR-novel-chr12_57357 | 5p | UGGGGG | 19 | GUGGGGGGCGGGGCGGACA |
| mmu-miR-novel-chr16_70802 | 5p | GAGGUA | 18 | GGAGGUAGUAGGUUGUGU |

Supplemental Table S3

*Predicted gene targets in affected PANTHER Pathways of upregulated neuronal miRNAs*

| **PANTHER Pathways** | **Filtered targets** | **Targeting miRNAs** |
| --- | --- | --- |
| **Hypoxia response via HIF activation** | Mtor | miR-101a-3p |
|  | Hif1a | miR-340-5p, miR-203-3p, miR-101a-3p |
|  | Pten | miR-142a-5p, miR-205-5p, miR-381-3p, miR-374b-5p |
|  | Akt3 | miR-101a-3p |
|  | Pik3r1 | miR-203-3p, miR-381-3p |
|  | Pik3c2a | miR-101a-3p, miR-381-3p |
|  | Egln1 | miR-203-3p |
|  | Egln3 | miR-142a-5p |
|  | Akt1 | miR-374b-5p |
|  | Pik3cd | miR-7a-5p |
|  | Akt2 | miR-203-3p |
|  | Arnt | miR-340-5p |
|  | Pik3r3 | miR-7a-5p |
|  | Pik3c2b | miR-101a-3p |
|  | Ngly1 | miR-381-3p |
|  | Pik3cb | miR-7a-5p |
|  | Pik3ca | miR-340-5p |
| **Axon guidance mediated by Slit/Robo** | Srgap1 | miR-142a-5p, miR-205-5p |
|  | Enah | miR-340-5p, miR-7a-5p |
|  | Slit2 | miR-381-3p |
|  | Ntng1 | miR-199b-5p, miR-205-5p |
|  | Robo1 | miR-340-5p, miR-142a-5p |
|  | Cxcr4 | miR-381-3p |
|  | Abl1 | miR-203-3p |
|  | Rac1 | miR-101a-3p |
|  | Slit3 | miR-340-5p, miR-374b-5p |
|  | Cdc42 | miR-340-5p, miR-381-3p |
|  | Dcc | miR-381-3p |
|  | Ntn1 | miR-340-5p, miR-381-3p, miR-374b-5p |
|  | Rhoc | miR-142a-5p |
|  | Robo2 | miR-101a-3p |

Supplemental Table S4

*Predicted gene targets in affected Reactome Pathways of upregulated neuronal miRNAs*

| **Reactome Pathways** | **Filtered targets** | **Targeting miRNAs** |
| --- | --- | --- |
| **Regulation of KIT signaling** | Socs6 | miR-340-5p, miR-203-3p, miR-381-3p |
|  | Socs1 | miR-142a-5p |
|  | Src | miR-203-3p |
|  | Kitl | miR-142a-5p, miR-101a-3p |
|  | Kit | miR-142a-5p |
|  | Fyn | miR-203-3p |
|  | Sh2b3 | miR-101a-3p |
|  | Sos1 | miR-374b-5p |
|  | Yes1 | miR-340-5p, miR-205-5p, miR-7a-5p, miR-381-3p |
|  | Cbl | miR-101a-3p |
|  | Lyn | miR-203-3p |
|  | Prkca | miR-340-5p, miR-203-3p |
| **Signaling by BMP** | Acvr2a | miR-203-3p, miR-101a-3p, miR-381-3p |
|  | Bmpr1a | miR-203-3p, miR-381-3p |
|  | Nog | miR-340-5p |
|  | Bmpr2 | miR-7a-5p |
|  | Zfyve16 | miR-203-3p, miR-205-5p, miR-381-3p |
|  | Smad1 | miR-203-3p |
|  | Bmpr1b | miR-101a-3p |
|  | Grem2 | miR-340-5p |
|  | Smurf1 | miR-203-3p |
|  | Ube2d1 | miR-340-5p, miR-142a-5p, miR-101a-3p |
|  | Smad6 | miR-374b-5p |
|  | Ube2d3 | miR-101a-3p, miR-381-3p |
|  | Bmp2 | miR-381-3p, miR-374b-5p |
|  | Ski | miR-340-5p |
|  | Smad4 | miR-340-5p, miR-381-3p |
| **Netrin-1 signaling** | Ezr | miR-205-5p |
|  | Neo1 | miR-1969, miR-374b-5p |
|  | Src | miR-203-3p |
|  | Trio | miR-205-5p, miR-101-3p |
|  | Nck1 | miR-340-5p |
|  | Myh10 | miR-423-5p, miR-381-3p |
|  | Rac1 | miR-101a-3p |
|  | Fyn | miR-203-3p |
|  | Unc5c | miR-340-5p, miR-205-5p |
|  | Cdc42 | miR-340-5p, miR-381-3p |
|  | Pitpna | miR-7a-5p |
|  | Ptk2 | miR-340-5p, miR-7a-5p, miR-381-3p |
|  | Dcc | miR-381-3p |
|  | Ntn1 | miR-340-5p, miR-381-3p, miR-374b-5p |
|  | Siah1a | miR-205-5p, miR-381-3p |
| **CD28 co-stimulation** | Mtor | miR-101a-3p |
|  | Src | miR-203-3p |
|  | Pik3r1 | miR-203-3p, miR-381-3p |
|  | Pdpk1 | miR-1969, miR-7a-5p, miR-374b-5p |
|  | Akt1 | miR-374b-5p |
|  | Map3k14 | miR-205-5p |
|  | Rac1 | miR-101a-3p |
|  | Fyn | miR-203-3p |
|  | Pak2 | miR-340-5p |
|  | Cdc42 | miR-340-5p, miR-381-3p |
|  | Mapkap1 | miR-7a-5p |
|  | Yes1 | miR-340-5p, miR-205-5p, miR-7a-5p, miR-381-3p |
|  | Stk4 | miR-381-3p, miR-374b-5p |
|  | Map3k8 | miR-101a-3p |
|  | Rictor | miR-340-5p, miR-203-3p, miR-381-3p |
|  | Cd28 | miR-142a-5p, miR-203-3p |
|  | Lyn | miR-203-3p |
|  | Pik3ca | miR-340-5p |
| **PKMTs methylate histone lysines** | Setd1a | miR-142a-5p |
|  | Rbbp5 | miR-1969 |
|  | Setd7 | miR-101a-3p |
|  | Aebp2 | miR-205-5p |
|  | Mecom | miR-7a-5p |
|  | Ezh2 | miR-101a-3p |
|  | Dpy30 | miR-101a-3p |
|  | Suz12 | miR-340-5p |
|  | Kmt2a | miR-205-5p |
|  | Dot1l | miR-101a-3p |
|  | Whsc1l1 | miR-340-5p |
|  | Rbbp7 | miR-101a-3p |
|  | Kmt2d | miR-1969 |
|  | Whsc1 | miR-101a-3p |
|  | Setd8 | miR-7a-5p |
|  | Setd2 | miR-142a-5p |
|  | Setd3 | miR-340-5p |
|  | Ehmt1 | miR-101a-3p |
|  | Eed | miR-101a-3p |
|  | Atf7ip | miR-340-5p, miR-203-3p, miR-205-5p, miR-101a-3p, miR-7a-5p |
| **Synthesis of PIPs at the plasma membrane** | Mtmr3 | miR-7a-5p |
|  | Pip4k2a | miR-205-5p |
|  | Pten | miR-142a-5p, miR-205-5p, miR-381-3p, miR-374b-5p |
|  | Synj1 | miR-340-5p, miR-142a-5p, miR-203-3p |
|  | Pik3r1 | miR-203-3p, miR-381-3p |
|  | Pip5k1c | miR-101a-3p |
|  | Pip4k2c | miR-1969 |
|  | Pik3c2a | miR-101a-3p, miR-381-3p |
|  | Inppl1 | miR-101a-3p, miR-7a-5p |
|  | Ocrl | miR-374b-5p |
|  | Pik3cd | miR-7a-5p |
|  | Pip5k1b | miR-142a-5p, miR-101a-3p, miR-381-3p |
|  | Pik3r3 | miR-7a-5p |
|  | Synj2 | miR-205-5p |
|  | Pik3c2b | miR-101a-3p |
|  | Pi4k2a | miR-203-3p |
|  | Pik3cb | miR-7a-5p |
|  | Pik3ca | miR-340-5p |

Supplemental Table S5

*Predicted gene targets in affected GO cellular components of upregulated neuronal miRNAs*

| **Cellular components** | **Filtered targets** | **Targeting miRNAs** |
| --- | --- | --- |
| **CCR4-NOT complex** | Cnot2 | miR-381-3p, miR-374b-5p |
|  | Patl1 | miR-1969, miR-7a-5p, miR-381-3p |
|  | Rqcd1 | miR-1969 |
|  | Cnot6l | miR-374b-5p |
|  | Zfp36 | miR-142a-5p |
|  | Tnks1bp1 | miR-1969 |
|  | Cnot4 | miR-381-3p |
|  | Cnot6 | miR-381-3p |
|  | Cpeb3 | miR-340-5p, miR-142a-5p, miR-101a-3p |
|  | Cnot7 | miR-381-3p |
|  | Cnot8 | miR-7a-5p |
|  | Cnot10 | miR-203-3p |
|  | Tob1 | miR-1969, miR-7a-5p, miR-381-3p |
| **cytoplasmic stress granule** | Larp4b | miR-374b-5p |
|  | Fmr1 | miR-101a-3p, miR-7a-5p, miR-374b-5p |
|  | Stau2 | miR-101a-3p |
|  | Larp1 | miR-205-5p, miR-381-3p, miR-374b-5p |
|  | Habp4 | miR-205-5p |
|  | Pum1 | miR-340-5p, miR-142a-5p, miR-203-3p, miR-205-5p, miR-381-3p |
|  | Ogfod1 | miR-7a-5p |
|  | Rc3h1 | miR-381-3p, miR-374b-5p |
|  | Lsm14a | miR-101a-3p, miR-381-3p |
|  | Eif4e | miR-203-3p, miR-205-5p, miR-1969, miR-7a-5p |
|  | Stau1 | miR-142a-5p, miR-203-3p |
|  | Zfp36 | miR-142a-5p |
|  | Pum2 | miR-340-5p, miR-142a-5p, miR-101a-3p, miR-1969, miR-381-3p |
|  | Caprin1 | miR-203-3p |
|  | Lin28a | miR-381-3p |
|  | Rptor | miR-1969 |
|  | Ddx3x | miR-340-5p, miR-101a-3p |
|  | Nufip2 | miR-203-3p, miR-205-5p |
|  | Ddx6 | miR-203-3p, miR-381-3p |
|  | Larp4 | miR-203-3p |
|  | Tia1 | miR-101a-3p |
|  | G3bp1 | miR-381-3p |
|  | Mbnl1 | miR-203-3p, miR-101a-3p, miR-381-3p |
|  | Tial1 | miR-203-3p, miR-205-5p |

Supplemental Table S6

*Predicted gene targets in affected PANTHER Pathways of downregulated miRNAs in lumbar motor neurons*

| **PANTHER Pathways** | **Filtered targets** | **Targeting miRNAs** |
| --- | --- | --- |
| **Histamine H_1_ receptor mediated signaling pathway** | Gng7 | miR-92b-5p |
|  | Prkch | miR-183-3p |
|  | Gng5 | miR-183-3p |
|  | Gna11 | miR-335-5p |
|  | Gnb1 | miR-183-3p |
|  | Prkcz | miR-148a-3p |
|  | Prkci | miR-183-3p |
|  | Prkce | miR-129-1-3p |
|  | Gng4 | miR-183-3p |
|  | Plcb1 | miR-148a-3p |
|  | Prkca | miR-183-3p |
| **Oxytocin receptor mediated signaling pathway** | Gng7 | miR-92b-5p |
|  | Prkch | miR-183-3p |
|  | Gng5 | miR-183-3p |
|  | Cacna1d | miR-129-1-3p |
|  | Gna11 | miR-335-5p |
|  | Gnb1 | miR-183-3p |
|  | Prkcz | miR-148a-3p |
|  | Prkci | miR-183-3p |
|  | Cacnb4 | miR-129-1-3p |
|  | Prkce | miR-129-1-3p |
|  | Gng4 | miR-183-3p |
|  | Plcb1 | miR-148a-3p |
|  | Prkca | miR-183-3p |
|  | Vamp2 | miR-127-3p |
| **PI3 kinase pathway** | Pten | miR-148a-3p |
|  | Irs1 | miR-183-3p |
|  | Ccnd2 | miR-183-3p |
|  | Gna11 | miR-335-5p |
|  | Gnb1 | miR-183-3p |
|  | Rps6kb1 | miR-148a-3p, miR-335-5p |
|  | Sos2 | miR-148a-3p |
|  | Pik3r3 | miR-148a-3p |
|  | Sos1 | miR-148a-3p |
|  | Gadd45a | miR-148a-3p |
|  | Nras | miR-148a-3p |
|  | Foxo1 | miR-183-3p |
| **Thyrotropin-releasing hormone receptor signaling pathway** | Gng7 | miR-92b-5p |
|  | Prkch | miR-183-3p |
|  | Gng5 | miR-183-3p |
|  | Gna11 | miR-335-5p |
|  | Gnb1 | miR-183-3p |
|  | Prkcz | miR-148a-3p |
|  | Prkci | miR-183-3p |
|  | Trh | miR-335-5p |
|  | Cacnb4 | miR-129-1-3p |
|  | Prkce | miR-129-1-3p |
|  | Gng4 | miR-183-3p |
|  | Plcb1 | miR-148a-3p |
|  | Prkca | miR-183-3p |
|  | Vamp2 | miR-127-3p |
| **AD-amyloid secretase pathway** | Bace2 | miR-335-5p |
|  | Mapk4 | miR-183-3p |
|  | Chrm2 | miR-129-1-3p |
|  | Prkch | miR-183-3p |
|  | Adam10 | miR-148a-3p |
|  | Mapk11 | miR-335-5p |
|  | Cacna1d | miR-129-1-3p |
|  | Psen2 | miR-183-3p |
|  | Prkcz | miR-148a-3p |
|  | Prkci | miR-183-3p |
|  | Mapk1 | miR-335-5p |
|  | Prkce | miR-129-1-3p |
|  | Furin | miR-129-1-3p |
|  | Prkca | miR-183-3p |
| **EGF receptor signaling pathway** | Ppp6c | miR-335-5p |
|  | Map3k1 | miR-129-1-3p, miR-183-3p |
|  | Ppp2ca | miR-183-3p |
|  | Prkch | miR-183-3p |
|  | Mapk11 | miR-335-5p |
|  | Map3k2 | miR-335-5p |
|  | Ar | miR-335-5p |
|  | Ppp2r5e | miR-125b-1-3p |
|  | Phldb2 | miR-183-3p |
|  | Ywhab | miR-148a-3p |
|  | Mras | miR-148a-3p |
|  | Hbegf | miR-183-3p |
|  | Prkcz | miR-148a-3p |
|  | Prkci | miR-183-3p |
|  | Map3k4 | miR-183-3p |
|  | Sfn | miR-127-3p |
|  | Mapk1 | miR-335-5p |
|  | Sos2 | miR-148a-3p |
|  | Sos1 | miR-148a-3p |
|  | Ppp2r5c | miR-183-3p |
|  | Cblb | miR-148a-3p |
|  | Erbb3 | miR-148a-3p |
|  | Rasal2 | miR-129-1-3p |
|  | Prkce | miR-129-1-3p |
|  | Rasa1 | miR-335-5p |
|  | Spry2 | miR-183-3p |
|  | Ppp2cb | miR-183-3p |
|  | Nras | miR-148a-3p |
|  | Prkca | miR-183-3p |
| **5-HT_2_ type receptor mediated signaling pathway** | Gng7 | miR-92b-5p |
|  | Prkch | miR-183-3p |
|  | Gng5 | miR-183-3p |
|  | Cacna1d | miR-129-1-3p |
|  | Gna11 | miR-335-5p |
|  | Gnb1 | miR-183-3p |
|  | Prkcz | miR-148a-3p |
|  | Prkci | miR-183-3p |
|  | Cacnb4 | miR-129-1-3p |
|  | Prkce | miR-129-1-3p |
|  | Gng4 | miR-183-3p |
|  | Plcb1 | miR-148a-3p |
|  | Prkca | miR-183-3p |
|  | Vamp2 | miR-127-3p |
| **FGF signaling pathway** | Ppp6c | miR-335-5p |
|  | Map3k1 | miR-129-1-3p, miR-183-3p |
|  | Ppp2ca | miR-183-3p |
|  | Prkch | miR-183-3p |
|  | Mapk11 | miR-335-5p |
|  | Map3k2 | miR-335-5p |
|  | Ppp2r5e | miR-125b-1-3p |
|  | Ywhab | miR-148a-3p |
|  | Fgf9 | miR-183-3p |
|  | Prkcz | miR-148a-3p |
|  | Prkci | miR-183-3p |
|  | Map3k4 | miR-183-3p |
|  | Sfn | miR-127-3p |
|  | Mapk1 | miR-335-5p |
|  | Ppp2r2a | miR-183-3p |
|  | Sos2 | miR-148a-3p |
|  | Sos1 | miR-148a-3p |
|  | Ppp2r5c | miR-183-3p |
|  | Prkce | miR-129-1-3p |
|  | Rasa1 | miR-335-5p |
|  | Spry2 | miR-183-3p |
|  | Ppp2cb | miR-183-3p |
|  | Nras | miR-148a-3p |
|  | Prkca | miR-183-3p |

Supplemental Table S7

*Predicted gene targets in affected Reactome Pathways of downregulated miRNAs in lumbar motor neurons*

| **Reactome Pathways** | **Filtered targets** | **Targeting miRNAs** |
| --- | --- | --- |
| **Post-transcriptional silencing by small RNAs** | Ago1 | miR-148a-3p |
|  | Tnrc6b | miR-125b-1-3p, miR-129-1-3p, miR-148a-3p |
|  | Ago4 | miR-148a-3p |
|  | Ago2 | miR-92b-5p, miR-148a-3p, miR-183-3p |
|  | Tnrc6c | miR-148a-3p |
|  | Tnrc6a | miR-148a-3p |
| **Signaling by TGF-beta Receptor Complex** | Tgfbr1 | miR-148a-3p |
|  | Smad2 | miR-148a-3p |
|  | Phip | miR-148a-3p |
|  | Arhgef18 | miR-183-3p |
|  | Cdk8 | miR-148a-3p |
|  | Usp9x | miR-125b-1-3p, miR-148a-3p |
|  | Ube2d1 | miR-148a-3p |
|  | Sp1 | miR-335-5p |
|  | Prkcz | miR-148a-3p |
|  | Skil | miR-183-3p |
|  | Ube2d3 | miR-148a-3p |
|  | Ccnt2 | miR-335-5p |
|  | Parp1 | miR-129-1-3p |
|  | Furin | miR-129-1-3p |
|  | Smurf2 | miR-148a-3p |
|  | Tgif2 | miR-148a-3p |
| **GAB1 Signalsome** | Pip4k2a | miR-125b-1-3p |
|  | Mtor | miR-92b-5p |
|  | Pten | miR-148a-3p |
|  | Ppp2ca | miR-183-3p |
|  | Irs1 | miR-183-3p |
|  | Pag1 | miR-148a-3p |
|  | Lck | miR-183-3p |
|  | Ppp2r5e | miR-125b-1-3p |
|  | Fgf9 | miR-183-3p |
|  | Hbegf | miR-183-3p |
|  | Kitl | miR-125b-1-3p |
|  | Mlst8 | miR-148a-3p |
|  | Mapk1 | miR-335-5p |
|  | Creb1 | miR-125b-1-3p, miR-129-1-3p |
|  | Cdkn1b | miR-148a-3p |
|  | Ppp2r5c | miR-183-3p |
|  | Erbb3 | miR-148a-3p |
|  | Rictor | miR-148a-3p |
|  | Chuk | miR-148a-3p |
|  | Ppp2cb | miR-183-3p |
|  | Foxo1 | miR-183-3p |
| **PI3K cascade: FGFR4,**  **PI3K cascade: FGFR3,**  **PI3K events in ERBB4 signaling,**  **PI3K cascade: FGFR2,**  **PI3K cascade: FGFR1,**  **PIP3 activates AKT signaling, PI3K/AKT activation** | Pip4k2a | miR-125b-1-3p |
|  | Mtor | miR-92b-5p |
|  | Pten | miR-148a-3p |
|  | Ppp2ca | miR-183-3p |
|  | Irs1 | miR-183-3p |
|  | Lck | miR-183-3p |
|  | Ppp2r5e | miR-125b-1-3p |
|  | Fgf9 | miR-183-3p |
|  | Hbegf | miR-183-3p |
|  | Kitl | miR-125b-1-3p |
|  | Mlst8 | miR-148a-3p |
|  | Mapk1 | miR-335-5p |
|  | Creb1 | miR-125b-1-3p, miR-129-1-3p |
|  | Cdkn1b | miR-148a-3p |
|  | Ppp2r5c | miR-183-3p |
|  | Erbb3 | miR-148a-3p |
|  | Rictor | miR-148a-3p |
|  | Chuk | miR-148a-3p |
|  | Ppp2cb | miR-183-3p |
|  | Foxo1 | miR-183-3p |

Supplemental Table S8

*Predicted gene targets in affected GO cellular components of downregulated miRNAs in lumbar motor neurons*

| **Cellular components** | **Filtered targets** | **Targeting miRNAs** |
| --- | --- | --- |
| **PP2A complex** | Ppp2ca | miR-183-3p |
|  | Ppp2r5e | miR-125b-1-3p |
|  | Ppp2r3a | miR-92b-5p |
|  | Ppp2r2a | miR-183-3p |
|  | Ppp2r5c | miR-183-3p |
|  | Ppp2r4 | miR-335-5p |
|  | Ppp2cb | miR-183-3p |
| **endocytic vesicle** | Rab8b | miR-183-3p |
|  | Dpp4 | miR-148a-3p |
|  | Syt11 | miR-335-5p |
|  | Uvrag | miR-183-3p |
|  | Rab14 | miR-148a-3p |
|  | Rapgef6 | miR-125b-1-3p, miR-335-5p |
|  | Unc13b | miR-183-3p |
|  | Ehd1 | miR-335-5p |
|  | Rabep1 | miR-127-3p |
|  | Lrp2 | miR-148a-3p, miR-183-3p |
|  | Sh3kbp1 | miR-335-5p |
|  | Amot | miR-148a-3p |
|  | Lck | miR-183-3p |
|  | Epn2 | miR-148a-3p |
|  | Lamp2 | miR-148a-3p |
|  | Rab9b | miR-183-3p |
|  | Rab34 | miR-148a-3p |
|  | Abca1 | miR-148a-3p, miR-183-3p |
|  | Rab11b | miR-335-5p |
|  | Snap91 | miR-148a-3p |
|  | Eps15 | miR-148a-3p |
|  | Nrxn1 | miR-335-5p |
|  | Clcn3 | miR-183-3p |
|  | Rab11fip4 | miR-183-3p |
|  | Ocln | miR-183-3p |
|  | Rala | miR-183-3p |
|  | Lamp1 | miR-125b-1-3p |
|  | Atg14 | miR-148a-3p |
|  | Picalm | miR-148a-3p, miR-183-3p |
